# Prognosis and therapeutic benefits prediction based on NK cell marker genes through single-cell RNA-seq with integrated bulk RNA-seq analysis for hepatocellular carcinoma

**DOI:** 10.3389/fonc.2023.1208165

**Published:** 2023-07-24

**Authors:** Yao Yang, Shaopin She, Liying Ren, Bigeng Zhao, Dongbo Chen, Hongsong Chen

**Affiliations:** ^1^ Peking University Hepatology Institute, Beijing Key Laboratory of Hepatitis C and Immunotherapy for Liver Diseases, Peking University People's Hospital, Beijing, China; ^2^ Laboratory of Hepatobiliary and Pancreatic Surgery, Affiliated Hospital of Guilin Medical University, Guilin, Guangxi, China

**Keywords:** hepatocellular carcinoma, natural killer cell, prognosis, immune infiltration, immunotherapy

## Abstract

Tumor-infiltrating immune cells greatly participate in regulating tumorigenesis and metastasis of hepatocellular carcinoma (HCC). Natural killer cell, as an important role of innate immunity, plays an indispensable role in antitumor immunity and regulate tumor development. In this study, we firstly identified 251 NK cell marker genes of HCC based on single-cell RNA sequencing data. Subsequently, an NK cell marker genes-related prognostic signature (NKPS) was developed in the cancer genome atlas (TCGA) cohort for risk stratification and prognosis prediction. The predictive value of the NKPS in prognosis was well validated in different clinical subgroups and three external datasets (ICGC-LIHC cohort, GSE14520 cohort and Guilin cohort). Moreover, multivariate analysis revealed the independent prognostic value of NKPS for OS in HCC. Further functional analysis indicated the NKPS was associated with basic cellular processes, that may contribute to the development and progression of HCC. Thereafter, immune characteristics as well as the therapeutic benefits in NKPS risk score-defined subgroups were analyzed. Patients with low-risk score exhibited immune-active status, manifested as higher immune scores, more infiltration of CD8+ T cells and macrophage M1, and higher T-cell receptor (TCR) richness and diversity. Remarkably, the NKPS was negatively correlated with immunotherapy response-related signatures. In addition, the low-risk group exhibited significantly improved therapeutic benefits, either from immunotherapy or traditional chemotherapy and target therapy. Overall, the NKPS showed an excellent predictive value for prognosis and therapeutic responses for HCC, which might also provide novel insights into better HCC management strategies.

## Introduction

Hepatocellular carcinoma (HCC), which comprises 75%-85% of primary liver cancer cases, is one of the most common primary malignant tumors with a fairly high incidence and poor prognosis (1, 2). Due to the occult onset, high degree of malignancy and fast progression of HCC, it is often diagnosed at an intermediate to advanced stage, at which point there is no chance for radical treatment (3). Besides, although great improvements have been achieved in the treatment strategy for HCC in recent years, HCC is prone to relapse and drug resistance, and ultimately leads to poor prognosis and high mortality. Therefore, developing a comprehensive and effective management strategy for HCC is urgently needed.

Several traditional clinical characteristics, such as TNM stage and AFP, have been widely proved to be associated with the prognosis of HCC patients, but they may not be sensitive and accurate enough to predict the prognosis of HCC let alone get involved in medical treatment decisions. The Barcelona clinic liver cancer (BCLC) system is one of the most widely and frequently used staging systems of HCC (4), but limited by the lack of information at the molecular level, the BCLC system performs inadequately in predicting long-term outcomes. Therefore, developing a model to effectively predict prognosis and therapeutic effects is crucial.

In recent decades, there is growing evidence indicating that immune cells in the tumor environment (TME) can exert either anti- or pro-tumor effects (5). Therefore, deeper insights into the roles of tumor-associated immune cells are important for exploring reasonable therapeutic strategies and improving the prognosis for HCC. As pivotal components of innate immunity, natural killer (NK) cells possess excellent cytolytic abilities independent of antigen stimulation, thus they constitute the first line of defense against tumors. NK cells can suppress tumor development directly by lysing tumor cells and indirectly by influencing the activities of other immune cells in the TME (6). Remarkably, increasing evidence indicated that tumor-infiltrating NK cells were less cytotoxic, because they expressed lower levels of granzyme B and CD57 and less IFN-γ (7, 8). On the other hand, the interaction of NK cells with tumor cells and stroma cells as well as cytokines in TME could lead to NK cell dysfunction. For example, immune checkpoints expressed on tumor cells and TGFβ1 secreted by tumor stromal cells could drive NK cell malfunction and may thus lead to increased tumor progression including invasion and metastasis (9–11). In addition, regulatory T cells and myeloid-derived suppressor cells can inhibit the activation and function of NK cells (12). Although the importance of NK cells in tumor control has been widely stressed, the contribution of tissue‐resident NK cells in HCC is not well understood. Thus, further investigation of the roles of NK cells in anti-tumor immunity at the molecular level is important for exploring reasonable therapeutic strategies and reducing HCC mortality.

Single-cell RNA-sequencing (scRNA-seq) technology has helped unravel molecular characteristics and biological processes of HCC (13, 14). Recent advancement in scRNA-seq technologies has enabled an in-depth and comprehensive exploration of expressional and functional states of various immune cells in TME. With the aid of scRNA-seq technologies, we identified a variety of NK cell marker genes and constructed a molecular signature to predict the long-term prognosis and therapeutic benefits of HCC patients. Subsequently, the performance of the signature was validated on various databases, and its related biological functions were explored.

## Methods

### Data collection

The Transcriptomic expression data and the corresponding clinical and survival information of HCC were collected from three different platforms, 365 HCC patients from the TCGA data portal (https://portal.gdc.cancer.gov/), 230 HCC patients from the ICGC data portal (https://dcc.icgc.org/projects/LIRI-JP), and 221 HCC patients from the Gene Expression Omnibus database (https://www.ncbi.nlm.nih.gov/geo/) (GSE14520). In addition, transcriptomic expression and clinical data from 67 HCC patients who received sorafenib and 147 HCC patients who were treated by TACE were downloaded from the GSE109211 dataset and GSE104580 dataset respectively. The transcriptomic expression and immunotherapy effect information of patients with melanoma treated with PD-1 inhibitor were obtained from GSE91061. The scRNA-Seq data for HCC were obtained from the China National Gene Bank Nucleotide Sequence Archive (*CNSA*: CNP0000650; https://db.cngb.org/cnsa).

Forty-eight patients diagnosed as primary HCC at the Affiliated Hospital of Guilin Medical University (Guilin, People’s Republic of China) between May 2002 and September 2010 were retrospectively included in this study (Guilin cohort). The Guilin cohort patients were diagnosed with HCC based on serological tests, radiological imaging, and pathological evaluations. Clinicopathologic information and tumor tissue of these 48 patients were collected.

### Processing single-sell RNA-seq data

Data for scRNA-seq analysis of 12 primary HCC samples were collected for this study. Cell filtering, classification, and visualization of the scRNA-seq data were analyzed by the R package “Seurat”. The top 2000 variable genes were used for further principal component analysis (PCA), and the T-distributed stochastic neighbor embedding (t-SNE) analysis was carried out. The “FindAllMarkers” function was applied to find marker genes for each cell cluster. Marker genes were selected as those with adjusted p values less than 0.01, average log_2_FC larger than 1. Cell clusters were annotated by the package “SingleR” and then checked manually.

### Establishment and validation of NK cell marker genes-related prognostic signature and nomogram construction

Univariate Cox regression analysis was used to identify NK cell marker genes associated with overall survival (OS) in HCC patients from the TCGA cohort. Significant OS-related genes were selected (P<0.01) to further perform variable selection using Least absolute shrinkage and selection operator (LASSO)-penalized Cox regression analysis. The significant predictors were selected using 1 standard error (1-SE) of the minimum criteria. Then, multivariate survival analysis was performed by stepwise Cox proportional hazards regression model to determine the most useful prognostic genes and then NK cell marker genes-related prognostic signature (NKPS) was constructed. The NKPS risk score of each patient was equal to the sum of the products of each gene’s normalized expression level and its corresponding regression coefficients. HCC patients were classified into high- or low-risk group according to the median value of risk score. The Kaplan–Meier algorithm was used to compare the OS or progression-free survival (PFS) between the two groups. Time-dependent receiver operating characteristic (ROC) curve was used to evaluate the accuracy of the prognostic model by the R package “survivalROC”. Subsequently, we performed subgroup analyses to validate the effectiveness of our prognostic signature in different clinical and pathological subgroups. Then, ICGC, GSE14520 and Guilin cohorts were applied for the external validations of the prognostic signature. In addition, univariate and multivariate cox regression analyses were performed to explore the correlation between the signature, clinical characteristics and OS. Finally, clinical characteristic parameters and the NKPS were adopted to establish a nomogram by the R package “rms”, to quantitatively investigate the probability of 1-, 3-, and 5-year OS of HCC patients. Subsequently, calibration curves were used to assess the consistency between predicted and actual survival outcome, and decision curve was used to assess the clinical net benefit of the nomogram.

### Functional enrichment analysis

Gene ontology (GO) and Kyoto Encyclopedia of Genes and Genomes (KEGG) pathway analyses were performed by the “clusterProfiler” package in R.

### Gene sets variation analysis

The gene set of immune processes was downloaded from the GSEA website (https://www.gsea-msigdb.org/gsea/msigdb/index.jsp). The Gene sets variation analysis (GSVA) was performed using the “GSVA” package in R. The functional enrichment score of each HCC sample was calculated and the enrichment result was visualized by the “pheatmap*”* package. The correlation between NKPS risk score and immune processes was determined by Pearson correlation analysis.

### Evaluation of immune infiltration and tumor immune microenvironment landscape

Infiltration of immune cells in the HCC sample was examined using the CIBERSORT algorithm with LM22 immune subsets(https://cibersort.stanford.edu/) (15). The “estimate” R package was used to calculate the immune cell infiltration level (immune score), stromal content (stromal score), comprehensive environmental score (ESTIMATE score) and tumor purity. Tumor transcriptome-based estimates of leukocyte fraction, lymphocyte infiltration signature, TGF-beta response, T cell receptor (TCR) richness and Shannon index, and tumor proliferation were obtained from the Pan-Cancer Atlas study of Thorsson et al. (16).

### Somatic mutation data processing

Somatic mutation data of HCC patients in the TCGA cohort was downloaded. The R package “maftools” was used to analyze, summarize, and visualize the somatic mutation data of patients in the TCGA cohort. Tumor mutational burden (TMB), a measure of the number of somatic mutations identified per megabase of DNA sequenced, and mutant allele tumor heterogeneity (MATH) score, a tumor-specific score based on the variation in variant allele frequency of all mutations in the tumor were calculated for every patient in the cohort. In addition, the differently mutated genes between the low-risk and high-risk groups were screened.

### Prediction of immunotherapeutic response and other therapeutic benefits of HCC patients based on NKPS

To assess the possible ability of risk score for prediction of immunotherapy response, firstly, the relationship between the risk score and immune checkpoint genes such as PD-L1, CTLA-4, LAG3, CD47 and TIM3 was explored. Zhu et al. reported that the atezolizumab + bevacizumab response signature (ABRS), which was derived from the genome-wide differential expression gene and GSEA analyses based on HCC patients who received atezolizumab + bevacizumab treatment in GO30140 and IMbrave150 studies, and T-effector signature (Teff) were highly associated with the clinical benefit of HCC patients to immune checkpoint inhibitors (ICIs) immunotherapy (17). Moreover, IFNG response and IFNA response signatures have been proven to be associated with response to ICIs. Therefore, we evaluated the correlation of the risk score with these ICI immunotherapy response-related signatures. In addition, TIDE score is a novel approach to evaluating the efficacy of ICIs immunotherapy, and it can be obtained from the TIDE website (http://tide.dfci.harvard.edu/) (18).

GSE91061 dataset included 109 melanoma cases with transcriptional expression and the efficacy of immunotherapy. According to the response to immunotherapy, patients in GSE91061 cohorts were classified into two subgroups: complete response (CR)+ partial response (PR), and stable disease (SD)+progressive disease (PD). We calculated the risk score of each patient and analyzed its impact on the prognosis and the efficacy of the PD-1 inhibitor. Furthermore, the predictive value of the NKPS risk score for chemotherapy benefit and sorafenib efficacy in HCC patients was evaluated based on GSE104580 and GSE109211 datasets, respectively.

### Real−time quantitative polymerase chain reaction

Total RNA of human HCC sample was extracted using TRIzol reagent (Invitrogen, USA) according to the manufacturer’s instructions and was reverse transcribed into cDNA using Hifair^®^ III 1st Strand cDNA Synthesis SuperMix for qPCR (YEASEN, China). The mRNA expression was assessed by real-time quantitative polymerase chain reaction (RT-qPCR) using Hieff UNICON ^®^ Universal Blue qPCR SYBR Green Master Mix (YEASEN, China). The relative mRNA expression levels of target genes were calculated by the comparative CT method. The primers used in this experiment are listed in **Supplementary Table S1**.

### Immunohistochemistry

The tissue slides of HCC sample through deparaffinization and dehydration were incubated with anti-CD8 primary antibody overnight at 4 °C after epitope retrieval, H_2_O_2_ treatment and non-specific antigens blocking. Slides were next incubated with secondary antibody, followed by signal detection with DAB staining kit (Zsbio, China)

### Statistical analysis

Data were presented as median (range). Medians were compared using the Wilcoxon rank-sum test. Chi-squared test or Fisher’s exact test was used to compare two percentages. The relationship between the risk score and other continuous variables was calculated by the Pearson method. Univariate and multivariate Cox regression analyses were implemented to identify independent predictors of OS. The OS and PFS between the different groups were evaluated by Kaplan-Meier analysis. If not specified above, a P value less than 0.05 was considered statistically significant, and all P values were two-tailed. All statistical analysis was performed in R 4.2.0.

## Results

### Identification of NK cell marker genes expression profiles

**Figure 1:**
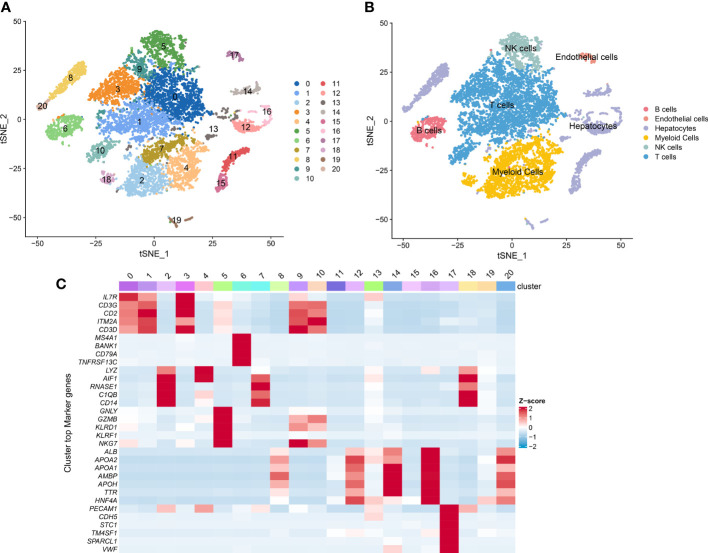
Single-cell RNA sequencing (scRNA-seq) analysis of HCC patients. **(A)** t-SNE visualization of the 10548 cells from 12 primary HCC tumor tissue in the CNP0000650 dataset. **(B)** t-SNE clustering of scRNA-seq colored by cell types. **(C)** Heatmap showing the expression of marker genes in each cluster.

We first obtained single-cell transcriptomic profile data of 12 primary HCC samples, consisted of 10548 single cells from the CNP0000650 dataset. PCA and t-SNE analysis was conducted to reduce the dimensionality by using the 2000 variable genes and identified 21 cell clusters (**Figure 1B**). According to expressions of marker genes, 6 major cell types were identified, including T cells, B cells, NK cells, myeloid cells, hepatocytes, and endothelial cells (**Figure 1A**). Heatmap showing the expression of marker genes in the indicated cell types (**Figure 1C**). Among the 21 distinct cell clusters, cells in cluster 5 were defined as NK cells and possess distinct gene expression profiles, with 251 differently expressed genes from other clusters. We identified these 251 genes as HCC-related NK cell marker genes. The GO enrichment and KEGG pathway analysis showed that these NK cell marker genes are highly enriched in immune-related processes (**Supplementary Figures S1A, B**).

### Construction of NK cell genes-related prognostic signature for the prognostic prediction of HCC

**Figure 2:**
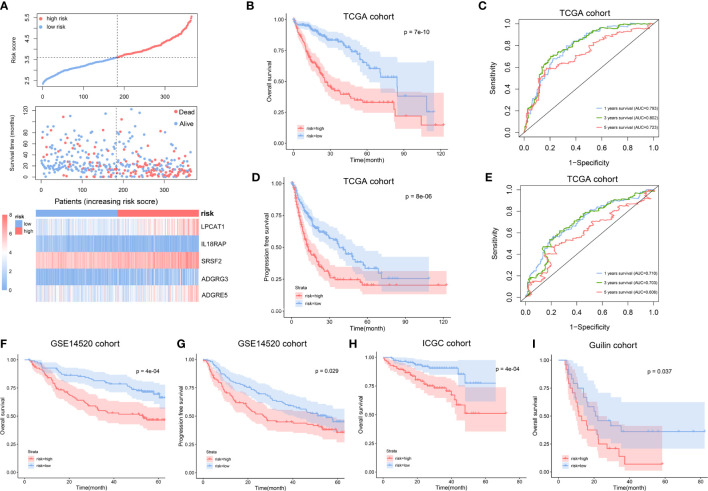
Construction and validation of NK cell marker genes-related prognostic signature (NKPS). **(A)** The distribution of risk scores and survival status, and the expression of the identified 5 NK cell marker genes in low- and high-risk groups. **(B)** Kaplan-Meier curve analysis of overall survival of HCC in low- and high-risk groups in TCGA cohort. **(C)** Time-dependent ROC analysis of the NKPS for predicting the risk of death at 1, 3, and 5 years in TCGA cohort. **(D)** Kaplan-Meier curve analysis of progression-free survival of HCC in low- and high-risk groups in TCGA cohort. **(E)** Time-dependent ROC analysis of the NKPS for predicting the risk of disease progression at 1, 3, and 5 years in TCGA cohort. **(F)** Kaplan-Meier curve analysis of overall survival of HCC in low- and high-risk groups in GSE14520 cohort. **(G)** Kaplan-Meier curve analysis of progression-free survival of HCC in low- and high-risk groups in GSE14520 cohort. **(H)** Kaplan-Meier curve analysis of overall survival of HCC in low- and high-risk groups in ICGC cohort. **(I)** Kaplan-Meier curve analysis of overall survival of HCC in low- and high-risk groups in Guilin cohort.

All 365 HCC patients with OS information in the TCGA cohorts were included to construct a prognostic model. We first performed univariate Cox regression analysis to explore NK cell marker genes related to OS, and 37 NK cell marker genes were found to be statistically significant (p < 0.01). After taking these 37 genes into the LASSO Cox regression model with minimized lambda, 12 NK cell marker genes were identified (**Supplementary Figure S2C**). To get a more practical model, we used stepwise cox proportional hazards regression to screen the most powerful predictive prognostic genes with regression coefficients. Five genes (LPCAT1, IL18RAP, SRSF2, ADGRG3, ADGRE5) (**Supplementary Figure S2D**) were identified to establish the NK cell marker genes-related prognostic signature (NKPS). The specific risk scores of OS were calculated as follows: risk score = (0.199* expression level of LPCAT1) + (-0.937* expression level of IL18RAP) + (0.492* expression level of SRSF2) + (0.189* expression level of ADGRG3) + (0.146* expression level of ADGRE5). HCC patients in the TCGA cohort were classified into low- and high-risk groups based on the median value of the risk score, and we compared the clinical and molecular differences between the two groups in the TCGA cohort (**Supplementary Table S2**). We found that a higher risk score was associated with more aggressive malignant characteristics, such as poorer tumor differentiation and more advanced tumor TNM stage. The distribution of risk score and survival status corresponding to the expression of each gene were displayed in **Figure 2A**. The Kaplan–Meier curve showed that patients in the high-risk group had significantly poorer OS than those in the low-risk group (p < 0.001) (**Figure 2B**). The areas under the curve (AUCs) of the time-dependent ROC curves for OS at 1, 3 and 5 years were 0.793, 0.802 and 0.723, respectively (**Figure 2C**). Moreover, the high-risk group had a significantly poorer PFS than the low-risk group (p < 0.001) (**Figure 2D**). The AUCs of the time-dependent ROC curves for PFS at 1, 3 and 5 years were 0.710, 0.703 and 0.606, respectively (**Figure 2E**). These results indicated high sensitivity and specificity of the NKPS for predicting OS and PFS.

### Validation of the NKPS in different clinical subgroups and external datasets

The prognostic value of NKPS was evaluated in different age, gender, TNM stage, pathological stage, AFP level, and HBV or HCV infection subgroups. The survival curve showed that in each subgroup, the patients with a high NKPS risk had significantly poorer OS compared to the patients with low NKPS risk (**Supplementary Figures S3A–L**).

To validate the prognostic performance of the NKPS in different data platforms, the GSE14520 dataset, ICGC dataset and Guilin cohort were used as external validation datasets. According to the same formula, the HCC patients in these three cohorts were classified into low-risk and high-risk groups using a median-risk score. We observed consistent differences in clinical characteristics between low-risk and high-risk groups (**Supplementary Table S2**). Consistent with the results in the TCGA cohort, the group with a high risk in the GSE14520 cohort showed significantly poorer OS and poor PFS relative to the group with a low risk (**Figures 2F, G**). The AUCs for 1-, 3-, and 5-year OS and 1-, 3-, and 5-year PFS were 0.677, 0.722, 0.658 and 0.664; 0.652, 0.603 respectively (**Supplementary Figures S4A, B**). Similarly, in the ICGC cohort, the high-risk group presented worse OS than those of the low-risk subgroup (p < 0.001) (**Figure 2H**). The AUC at 1-, 3- and 5- years were 0.762, 0.723, 0.715, respectively (**Supplementary Figure S4C**). Moreover, in accordance with the results in the public dataset, a higher survival rate was observed in low-risk group in our Guilin cohort (p=0.037) (**Figure 2I**).

### Independent prognostic value of NKPS

To portray a detailed prognostic value of the NKPS, we performed univariate and multivariate Cox regression analysis in TCGA, ICGC, GSE14520, and Guilin cohorts, respectively. In univariate Cox regression analyses, the risk score was significantly associated with OS in all four HCC cohorts. Moreover, after correction for other confounding factors by multivariate Cox regression, the NKPS was still significantly related to OS in all four HCC cohorts (TCGA cohort: HR = 2.57, 95% CI = (1.99, 3.33), p<0.001; ICGC cohort: HR = 2.06, 95% CI = (1.34,3.15), p = 0.001; GSE14520 cohort: HR = 2.06, 95% CI = (1.25, 3.40), p<0.001; Guilin cohort: HR = 1.87 95% CI = (1.20, 2.80), p=0.003), which indicated that NKPS could independently predict the prognosis in each cohort (**Table 1**).

**Table 1:** Univariate and multivariate Cox regression analyses of variables related to OS in the TCGA, ICGC, GSE14520 and Guilin cohorts.

	TCGA cohort				ICGC cohort				GSE14520 cohort				Guilin cohort			
Characteristics	Univariable analysis		Multivariable analysis		Univariable analysis		Multivariable analysis		Univariable analysis		Multivariable analysis		Univariable analysis		Multivariable analysis	
	HR (95% CI)	p	HR (95% CI)	p	HR (95% CI)	p	HR (95% CI)	p	HR (95% CI)	p	HR (95% CI)	p	HR (95% CI)	p	HR (95% CI)	P
**Age**	1.01 (1.00,1.03)	0.07			1.00 (0.97,1.03)	0.81			0.99 (0.97,1.01)	0.40			1.05 (0.67,3.93)	0.28		
Gender																
female	1 (ref)				1 (ref)		1 (ref)		1 (ref)				1 (ref)		1 (ref)	
male	0.81 (0.57,1.16)	0.26			0.50 (0.27,0.95)	0.03	0.28 (0.13,0.59)	0.001	1.7 (0.82,3.52)	0.15			1.39 (0.54,3.59)	0.50		
TNM stage																
I/II	1 (ref)		1 (ref)		1 (ref)		1 (ref)		1 (ref)		1 (ref)		1 (ref)			
III/IV	2.54 (1.79,3.61)	<0.001	2.06 (1.44,2.94)	<0.001	2.47 (1.34, 4.57)	0.004	2.60 (1.27,5.30)	0.009	3.52 (2.24, 5.51)	<0.001	2.73 (1.70,4.38)	<0.001	2.13 (1.06,4.27)	0.033	1.58 (1.02,2.45)	0.042
Grade																
1/2	1 (ref)				1 (ref)											
3/4	1.12 (0.78,1.61)	0.54			2.61 (1.37, 4.99)	0.004	2.12 (1.09,4.13)	0.026								
**AFP**																
low	1 (ref)								1 (ref)		1 (ref)					
high	1.03 (0.64,1.64)	0.92							1.69 (1.06, 2.50)	0.03	1.18 (0.75,1.85)	0.49				
BCLC stage																
0/A									1 (ref)							
B/C									3.54 (2.27,5.54)	<0.001						
**Risk score**	2.72 (2.12,3.49)	<0.001	2.57 (1.99,3.33)	<0.001	2.28 (1.55,3.32)	<0.001	2.06 (1.34,3.15)	0.001	2.41 (1.54,3.78)	<0.001	2.06 (1.25,3.40)	0.005	2.16 (1.40,3.34)	<0.001	1.87 (1.20,2.80)	0.003

HR, hazard ratio; CI, confidence interval; AFP, alpha-fetoprotein; TNM, tumor node metastasis; BCLC, Barcelona clinical liver cancer.

### NKPS-related biological functions

**Figure 3:**
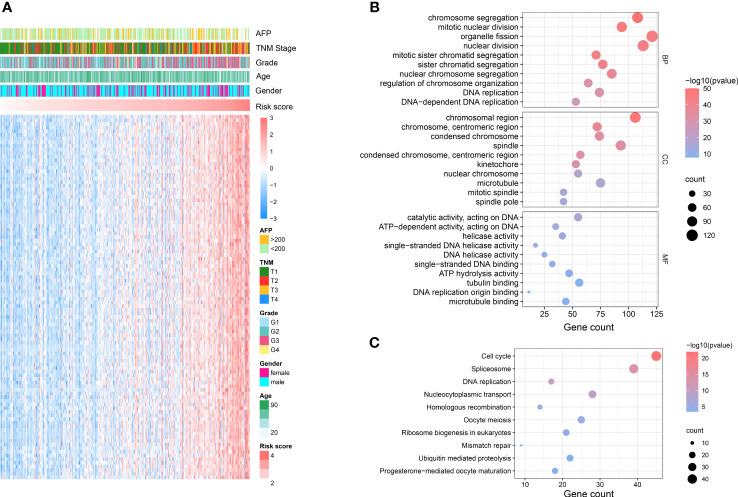
NKPS related biological functions. **(A)** Heatmap showed the top 100 genes that had the most significant correlations with NKPS. (Pearson R> 0.5, P < 0.001). **(B)** Representative GO terms of the correlated genes. **(C)** Representative KEGG enrichment results of the correlated genes.

To explore the potential mechanism of how NKPS predicts HCC prognosis, we investigated the biological function of NKPS. Firstly, genes highly related to NKPS were identified in the TCGA cohort with Pearson correlation coefficient >0.5 and p<0.001, and 1064 positively-correlated genes were identified. The top 100 positively correlated genes were plotted in a heatmap (**Figure 3A**). Next, GO analysis and KEGG analysis were conducted. GO analysis revealed that these genes were mostly correlated to basic cellular processes, including chromosome segregation, nuclear division, DNA replication, mRNA processing, translation and cell proliferation (the top5 GO enrichment results in each category were shown in **Figure 3B**). KEGG pathways analysis also reflected high enrichment of pathways involved in the cell proliferation, including cell cycle and DNA replication (the top10 KEGG enrichment results were shown in **Figure 3C**).

### Association between the NKPS and immune cell infiltration in the tumor microenvironment

**Figure 4:**
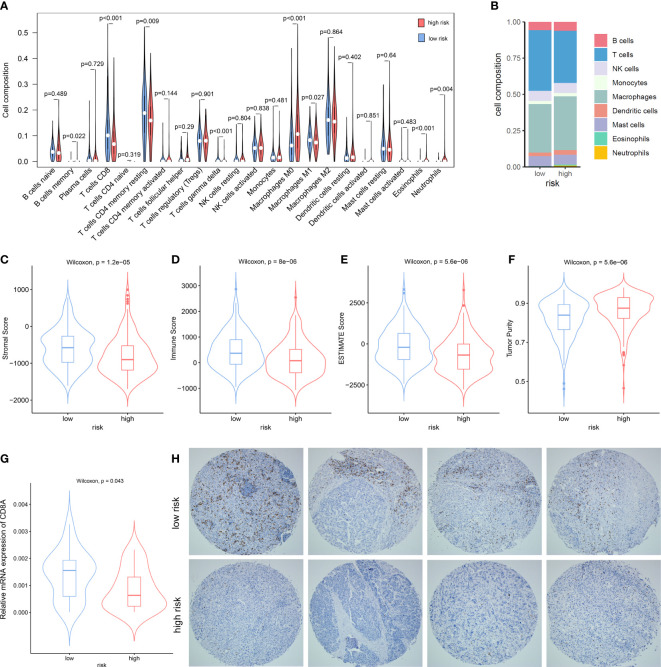
The Association between the NKPS and immune cell infiltration in tumor microenvironment. **(A)** The comparison of immune cells infiltration level of 22 immune cell types between low-risk and high-risk groups. **(B)** The composition of different immune cells between low- and high-risk groups. **(C–F)** comparison of stromal score **(C)**, immune score **(D)**, ESTIMATE score **(E)** and Tumor purity **(F)** between low-risk and high-risk groups. **(G)** The relative mRNA expression of CD8A in the Guilin cohort. **(H)** Representative immunostaining pictures of CD8 between low-risk and high-risk groups in the Guilin cohort.

Since anti-tumor immunity is highly modulated by the proliferation and activation of immune cells, we explored the association between the NKPS and immune cell infiltration in the tumor microenvironment in HCC patients. We characterized and analyzed the infiltrating level of different immune cells in low- and high-risk HCC patient groups based on NKPS. CIBERSORT-based analysis revealed that low-risk patients have significantly higher CD8+ T cell, T cell CD4 memory resting, T cells gamma delta, and significantly lower M0 macrophage, eosinophils and neutrophils compared to the high-risk group (**Figure 4A**). Composition of tumor-infiltrating immune cells in two groups was presented in **Figure 4B**. Moreover, *via* ESTIMATE algorithm, we observed that the high-risk group had higher tumor purity than the low-risk group (**Figure 4F**), while the low-risk group exhibited higher stromal score, immune score and ESTIMATE score (**Figures 4C–E**).

Since the killing of tumor cells by CD8+ T cells is the principal mechanism of immune protection against tumors, and the difference in CD8+ T cells infiltration between the high-risk and low-risk group in our study was most striking, we detected the infiltration level of CD8+ T cells in tumor tissues by RT-PCR and immunohistochemistry. In accordance with the immune infiltration analysis, proportions of CD8+ T cells infiltrated in low-risk tumors were significantly higher than those in high-risk group (p = 0.043) (**Figure 4G**). Representative immunohistochemistry staining pictures of high- and low-risk tumors were showed in **Figure 4H**.

### Distinct immune response and inflammatory profiles in tumors among NKPS subgroups

**Figure 5:**
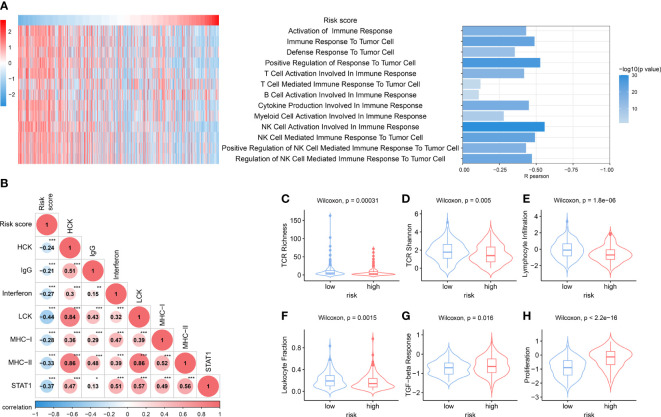
Distinct immune response and inflammatory profiles in tumors among NKPS subgroups. **(A)** the heatmap showed the risk score and the enrichment scores of immune functions of each patient in the TCGA cohort. The bar plot on the right showed the R- value and P- value of the correlation analysis. **(B)** Correlation matrix of risk score and seven immune and inflammatory-related metagenes based on TCGA cohort. The number inside the circle represents the R- value of the correlation analysis. Asterisks indicates significance of the correlation: one asterisk, P<0.05; two asterisks, P<0.01; three asterisks, P<0.001. **(C–H)** Boxplots for various immune response-related scores among low-risk and high-risk group in the TCGA cohort. **(C)** TCR richness, **(D)** TCR Shannon, **(E)** lymphocyte infiltration, **(F)** leukocyte fraction, **(G)** TGF-beta response, **(H)** proliferation.

We then further explored the correlation between NKPS with immune function. Immune-associated pathway enrichment score of each sample in the TCGA cohort was calculated by GSVA. We found that NKPS was significantly negatively associated with enrichment scores of immune-associated pathways, including activation of different immune cells involved in immune response, cytokine production involved in immune response, and various immune cells mediated immune response to tumor cell (**Figure 5A**). Moreover, to get a further understanding of NKPS-related inflammatory and immune activities, we collected seven inflammatory and immune-related gene signatures, including HCK, IgG, LCK, MHC-I, MHC-II, STAT1 and interferon. **Figure 5B** showed that NKPS was negatively correlated with these seven inflammatory and immune-related gene signatures. Regarding cancer immunity scores provided by Thorsson et al. for the TCGA cohort (16), the low risk group had higher scores of leukocyte fraction, lymphocyte infiltration signature, macrophage regulation, T cell receptor (TCR) Richness and TCR Shannon index, while the lower score of TGF-beta response and lower level of proliferation compared to high-risk score (**Figures 5C–H**).

### Different somatic alteration landscapes between the high− and low−risk groups

**Figure 6:**
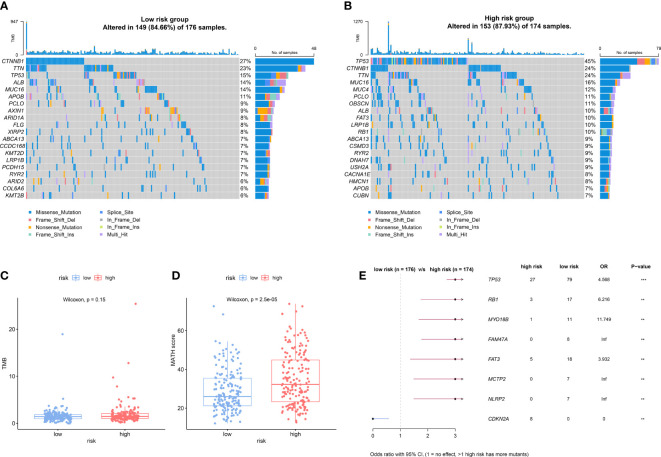
Different somatic alteration landscape between the low- and high-risk groups. **(A, B)** The Oncoplot was constructed by the top 20 mutation genes in the low-risk **(A)** and high-risk **(B)** subgroups. Each liver tumor from an individual patient in TCGA cohort was represented in each column. **(C, D)** The difference of Tumor mutation burden (TMB) **(C)** and mutant allele tumor heterogeneity (MATH) score **(D)** in low- and high-risk groups. **(E)** The forest plot shows the most significantly differently mutated genes between the low- and high-risk group. ** indicating p<0.01; *** indicating p<0.001.

To explore the somatic alteration landscape between the low- and high-risk groups, we analyzed somatic mutation data of HCC patients in the TCGA cohort. Although there was no significant difference in TMB between the low- and high-risk groups (**Figure 6C**), the high-risk group presented a significantly higher MATH score (**Figure 6D**), which indicated a higher inter-tumor heterogeneity. The top 20 variant mutations in the low- and high-risk groups were displayed in **Figures 6A, B**, and the forest plot showed the mostly differentially mutated genes between the two groups (**Figure 6E**). The TP53 gene mutation differed most significantly between the two groups (mutation rate of the high-risk group vs the low group: 47% vs 14%, OR, 5.234, p<0.001).

### Prediction of immunotherapy response and other therapeutic efficacy based on NKPS

**Figure 7:**
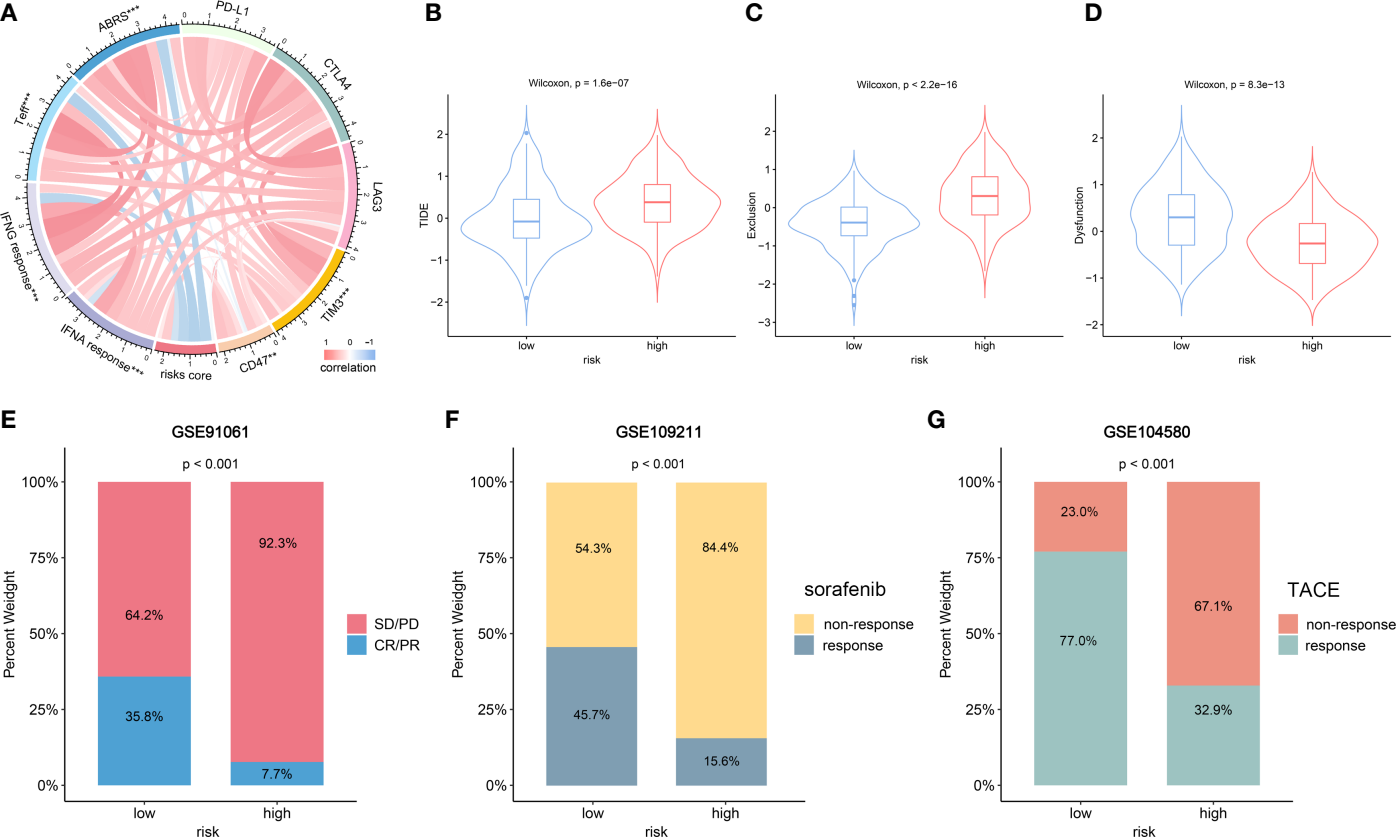
The role of NKPS in predicting immunotherapeutic response and other therapeutic benefits for HCC patients. **(A)** The correlation between risk score and inhibitory immune checkpoints and signatures which can predict the response to immunotherapy. The color of the band represented the Pearson R-value. **(B–D)** Tumor immune dysfunction and exclusion (TIDE) score **(B)**, T cell exclusion score **(C)** and T cell dysfunction score **(D)** between low-risk and high-risk groups. **(E)** Treatment response rates of anti-PD-1 immunotherapy in low- and high -risk groups in the GSE91061 cohort. **(F)** Treatment response rates of sorafenib in low-risk and high-risk groups in the GSE109211 cohort. **(G)** Treatment response rates of TACE in low-risk and high-risk groups in the GSE104580 cohort.

We investigated the relationship between NKPS risk score and five immune checkpoint molecules (PD-L1, CTLA4, LAG3, TIM3 and CD47). The risk score showed a positive relationship with the expression of TIM3 and CD47 (**Figure 7A**). Next, we examined the correlation between the risk score and several signatures that are closely related to the response to ICIs in HCC patients. As shown in **Figure 7A**, the NKPS were significantly inversely related to ABRS, Teff, IFNA response and IFNG response (all p<0.001). In addition, TIDE algorithm was applied to predict the likelihood of immunotherapy response of each HCC patient in the TCGA cohort. The results showed that the low-risk group possessed significantly higher TIDE and exclusion scores and lower dysfunction scores compared to the low-risk group (**Figures 7B–D**). Above results suggested that HCC patients in the low-risk group are more likely to be responsive to ICI therapy. Due to a lack of suitable publicly available transcriptional data about HCC patients who underwent ICI treatment, we collected the GSE91061 dataset, which included 109 anti-PD-1 treated malignant melanoma samples, to validate the potential predictive value of NKPS. The results showed more ICI responders (CR/PR: complete response/partial response) were enriched in the low‐risk subgroup (35.8% vs 7.7%, p<0.001) (**Figure 7E**).

Sorafenib and TACE are the two commonly used therapeutic modalities for HCC patients, and we also evaluated the predictive value of NKPS in HCC patients who received sorafenib and TACE therapy. Bar plot (**Figure 7F**) showed a significantly higher response rate of sorafenib in the low-risk group than in the high-risk group (44.1% vs 18.2%, p=0.022). Similarly, more individuals in the low-risk group presented a response to TACE than that in the high-risk group (77.0% vs 32.9%, p<0.001) (**Figure 7G**).

### Construction of a nomogram based on the NKPS

**Figure 8:**
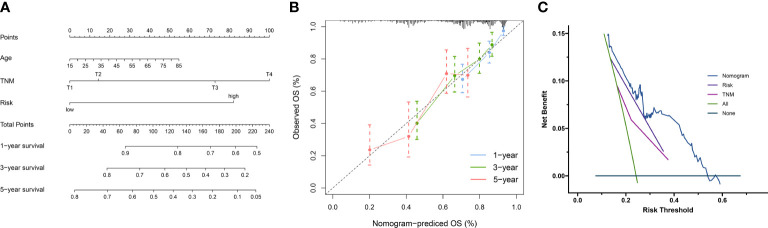
**(A)** Nomogram combining clinical characteristic parameters and NKPS risk score for predicting overall survival for HCC patients. **(B)** The predicted calibration curve approached the standard curve at the 1-, 3- and 5-year calibration points. **(C)** Decision curve of the nomogram.

NKPS was proven to be a significantly independent prognostic factor through Cox regression analysis with multiple clinical features (**Table 1**). To quantitatively evaluate the survival probability of HCC individuals in the clinical setting, we combined it with other clinicopathological traits to construct a nomogram (**Figure 8A**).

The C-index of 0.73 indicated the good performance of nomogram. Furthermore, the calibration curve shown good agreement between predicted and the actual probability at different survival time points, including 1-, 3-, and 5-year OS (**Figure 8B**). The decision curve showed that the nomogram had the best predictive performance (**Figure 8C**)

## Discussion

NK cells, which make up roughly 50% of the hepatic lymphocyte pool (19), play an indispensable role in tumor surveillance and control. Studies have shown that intrahepatic NK cells possess higher cytotoxic activity against tumor cells than peripheral blood NK cells (20). In addition, Xue et al. reported that high NK cell levels could predict better survival for patients with HCC (21). Thus, a more comprehensive understanding of the role of NK cells in HCC would undoubtedly contribute to better surveillance and management of HCC.

In this study, we firstly used scRNA-seq for a comprehensive view of transcriptional profile of the intrahepatic NK cells. Subsequently, NKPS, a robust prognostic signature for HCC based on NK cell marker genes was established and well-validated. Patients with low-risk NKPS scores in the TCGA cohort showed better OS and RFS. Besides, similar results were observed in three independent validation cohorts, ICGC, GSE14520 and Guilin cohorts. Moreover, when performing a subgroup analysis, the prognostic difference between the low-risk and high-risk groups was observed in different clinical characteristics. Multivariate Cox regression analyses in the three cohorts suggested the independent role of NKPS as a predictive marker of the long-term prognosis of HCC. These results indicated the effectiveness and robustness of NKPS in predicting HCC prognosis.

The NKPS proposed in the present study was composed of 5 NK cell marker genes (LPCAT1, IL18RAP, SRSF2, ADGRG3, ADGRE5). The IL18RAP gene, encoding an indispensable subunit of the IL-18R complex, plays an important role in IL18 signaling transduction (22). The prognostic role of IL18RAP has been reported in various cancers, including HCC, renal cell carcinoma and esophageal carcinoma (23–25). The proteins encoded by ADGRE5 and ADGRG3 belong to the family of adhesion G protein-coupled receptors, which could modulate the cellular processes closely related to tumor cell biologies, such as cell adhesion and detachment, migration, polarity, and guidance (26, 27). Studies have shown that the expression of ADGRE5 correlated with tumor cell invasion and angiogenesis, leading to a poor clinical prognosis in several cancers (28, 29). LPCAT1, as a key enzyme regulating phospholipid metabolism, could modulate cell membrane fluidity and facilitate tumor cell metastases in HCC (30). Splicing factor SRSF2 acts as a critical regulator for cell survival, its mutation or dysregulation of expression has been reported to drive the process of hepatocarcinogenesis (31, 32). This study identified five genes as a prognostic molecular signature, which may also provide an increased understanding of the molecular mechanisms underlying the pathogenesis of HCC.

We further performed a series of functional analyses to explore the possible mechanism behind the prognostic power of the NKPS. GO and KEGG analyses based on the genes closely related to the NKPS revealed that these related genes were mostly associated with cellular proliferation, cell cycle and DNA replication. Since NK cell can directly induce apoptosis in tumor cells or induce ADCC activity to lyse tumor cells, dysfunction of NK cells can cause dysregulation of cell cycle and cellular proliferation, resulted in unrestrained cell growth and tumor development, and then leading to a poor prognosis. In addition, the genetic landscape analysis found that high-risk score patients exhibited significantly higher inter-tumor heterogeneity. Remarkably, the mutation of several tumor suppressor genes, such as TP53 and RB1, occurred more frequently in patients with high-risk scores. Previous studies have shown that mutations of TP53 and RB1 were correlated with uncontrolled cell cycle progression, and contributed to poor survival in HCC (33, 34). These results hinted the abnormal cellular process in patients with high-risk scores and might explain the more aggressive tumor characteristics and worse prognosis of high-risk patients

Furthermore, we investigated the relationship between tumor immune cell infiltration and NKPS. The estimate algorithm demonstrated the negative correlation between NKPS and immune infiltration. Patients with high NKPS risk scores possessed lower immune scores, stromal scores and ESTIMATE scores, whereas higher tumor purity, as compared to patients with a low NKPS risk score. Specifically, we also evaluated the relative infiltration of immune cells in high-risk and low-risk groups. The results showed that patients with low-risk scores were in an immune-active state, manifested as high infiltration of CD8+ T cells and macrophages M1. In contrast, patients with high-risk scores possessed a higher frequency of macrophage M0, neutrophils and eosinophils, which have been proved implicated in immunosuppression and tumor progression (35, 36). These results could explain the predictive capacity of the NKPS. CIBERSORT-based analysis showed that the difference of infiltration level of NK cells between NKPS high and low groups was not obvious, possible reasons include the following, firstly, all genes used in preliminary screening were NK cell marker genes, and secondly, we performed lasso-cox regression to determine the critical genes in HCC patients’ survival and then constructed the prognostic model, so patients in the NKPS high and low groups differed mostly by long-term survival. Although the infiltration level of NK cells between NKPS high and low groups was not so obvious, immune-associated pathway enrichment analysis showed that patients with high NKPS risk score possess less score of NK cell anti-tumor function, such as low score of NK cell activation involved in immune response and NK cell mediated response to tumor cell.

Furthermore, GSVA showed that NKPS was negatively correlated with immune and inflammatory responses. Moreover, patients in the low-risk group presented higher TCR richness and diversity, which implied an improved ability of antigen uptake and presentation. In addition, the host defense immunity activity (leukocyte fraction, and lymphocyte infiltration) was higher in the low-risk group compared with the high-risk group. Moreover, we validated the higher infiltration of CD8+ T cells in the low-risk group based on our Guilin cohort. These results showed that the low-risk group was in immune-active status. Meanwhile, these results revealed that the potential mechanism of the prognostic ability of NKPS may be related to the immune responses.

The recent advent of immunotherapy, especially immune checkpoint inhibitors (ICIs), has dramatically transformed the treatment landscape for advanced HCC, but not all patients could benefit from immunotherapy. Therefore, exploring a potential prognostic biomarker to identify patients who would benefit the most from ICIs is crucial. In this study, the correlation analyses suggested that the NKPS was significantly negatively correlated with the ICI immunotherapy response-related signatures, including ABRS, Teff, IFNA response and IFNG response signature, which means patients with low-risk scores will benefit more from immunotherapy. Moreover, TIDE is a newly discovered immunotherapy predictor and has been proven to have better predictive performance than other biomarkers or indicators (18). A higher TIDE score represented a higher potential for immune evasion, which indicated that the patients were less likely to benefit from ICI therapy. In this study, the low-risk subgroup had a lower TIDE score than the high-risk subgroup, implying that NKPS-low patients could benefit more from ICI therapy than NKPS-high patients. Due to the lack of publicly available large-scale sequencing data of HCC patients receiving immunotherapy. Meanwhile, aiming to investigate the wider applicability of NKPS, melanoma patients receiving anti-PD-1 therapy were enrolled. A significantly higher response rate (CR/PR) in the low-risk subgroup validated the predictive power of NKPS for immunotherapeutic effect. Besides, transcatheter arterial chemoembolization (TACE) and sorafenib are two of the major and widely used treatment methods for advanced HCC. In our results, patients with low risk scores were more likely to respond to sorafenib or TACE than patients with high risk scores. Taken together, NKPS could act as a reliable biomarker for predicting response to immunotherapy or traditional therapies, therefore, it’s helpful to formulate strategies to better manage HCC patients.

There did exist some limitations in this study. Firstly, the prognostic signature was constructed based on the data from the public datasets, additional verification by large-scale clinical trials is needed. Furthermore, these findings need to be validated and explored in immunotherapy trials. Secondly, although our study indicated that NKPS can be used to predict response to immunotherapy of HCC patients, validation in independent cohorts of HCC patients who were treated with immunotherapy is required. Lastly, the specific mechanisms underlying the NKPS’s prognostic power require further experimental verification and discussion.

In conclusion, we constructed and validated a prognostic signature consisting of five NK cell marker genes for HCC based on scRNA-seq and bulk RNA-seq analysis. The NKPS possesses an excellent ability to distinguish the malignant degree of the tumor and the prognosis of patient. In addition, NKPS could be used as a powerful tool to predict the therapeutic benefits of HCC and provide treatment guidance for HCC.

## Data availability statement

The original contributions presented in the study are included in the article/**Supplementary Material**. Further inquiries can be directed to the corresponding authors.

## Ethics statement

The studies involving human participants were reviewed and approved by the Ethics Committee of the Affiliated Hospital of Guilin Medical University and Peking University People’s Hospital. The patients/participants provided their written informed consent to participate in this study.

## Author contributions

YY, DC and HC were responsible for study design, critical revision of the manuscript and obtaining funding. YY and LR analyzed data, interpreted results. YY, SS and BZ performed the experiments. YY and SS wrote and revised the manuscript. All authors contributed to the article and approved the submitted version.

## Supplementary material

Supplementary Figure 1 Functional enrichment analysis of NK cell marker genes of HCC. **(A)** Representative GO terms of the NK cell marker genes. **(B)** Representative KEGG enrichment results of the NK cell marker genes.

Supplementary Figure 2 Construction of NK cell marker genes-related prognostic signature in TCGA cohort. **(A)** LASSO coefficient profiles of the 37 prognostic genes. **(B)** In LASSO regression analysis, partial likelihood deviance plot showed cross-validation for tuning parameter screening. **(C)** Forest plots showing the results of the univariate Cox regression analysis between the 12 genes expression (identified by LASSO regression analysis) and OS in the TCGA cohort. **(D)** Step Multivariable Cox proportional hazards regression analysis of the 5 prognostic genes.

Supplementary Figure 3 Validation of the prognostic performance of NKPS in different clinical subgroups. Kaplan–Meier curves of overall survival in male **(A)**, female **(B)**, young **(C)**, old **(D)**, TNM I/II **(E)**, TNM III/IV **(F)**, early stage **(G)** and advanced stage (pathological grade 3/4) **(H)**, AFP ≥200ng/ml **(I)**, AFP<200 **(J)**, HBV **(K)** and HCV **(L)** patients based on risk score in TCGA cohort.

Supplementary Figure 4 Time-dependent ROC analysis of the NKPS for predicting the risk of death in different cohorts. **(A)** Time-dependent ROC analysis of the NKPS for predicting the risk of death at 1, 3, and 5 years in GSE14520 cohort. **(B)** Time-dependent ROC analysis of the NKPS for predicting the risk of disease progression at 1, 3, and 5 years in GSE14520 cohort. **(C)** Time-dependent ROC analysis of the NKPS for predicting the risk of death at 1, 3, and 5 years in ICGC cohort. **(D)** Time-dependent ROC analysis of the NKPS for predicting the risk of death at 1, 3, and 5 years in Guilin cohort.
