# Supplementary figures and images for: Prognosis and therapeutic benefits prediction based on NK cell marker genes through single-cell RNA-seq with integrated bulk RNA-seq analysis for hepatocellular carcinoma
Source: Front Oncol. 2023 Jul 24;13:1208165. doi: 10.3389/fonc.2023.1208165 (PMC10406383; doi:10.3389/fonc.2023.1208165)

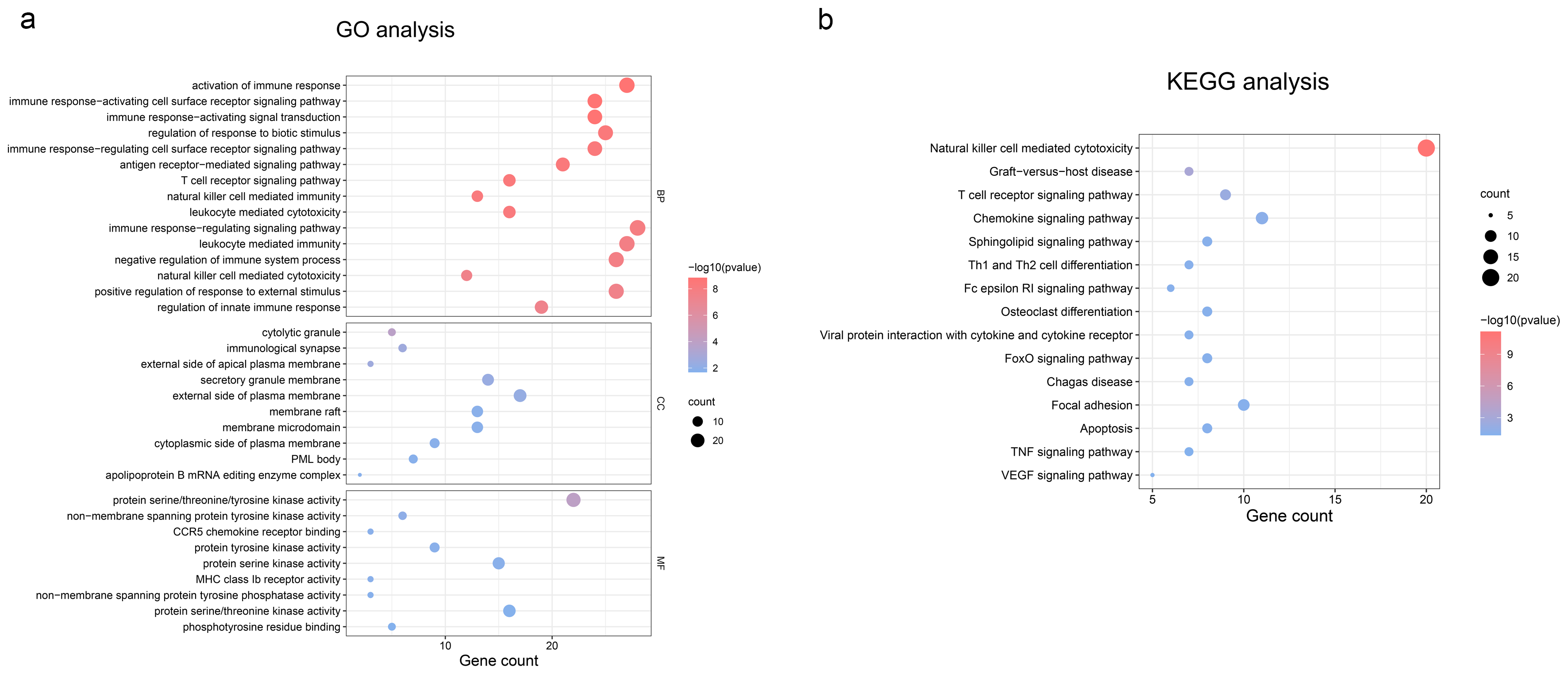

Supplement: Supplementary Figure 1 — Functional enrichment analysis of NK cell marker genes of HCC. (A) Representative GO terms of the NK cell marker genes. (B) Representative KEGG enrichment results of the NK cell marker genes. [file Image_1.tif]

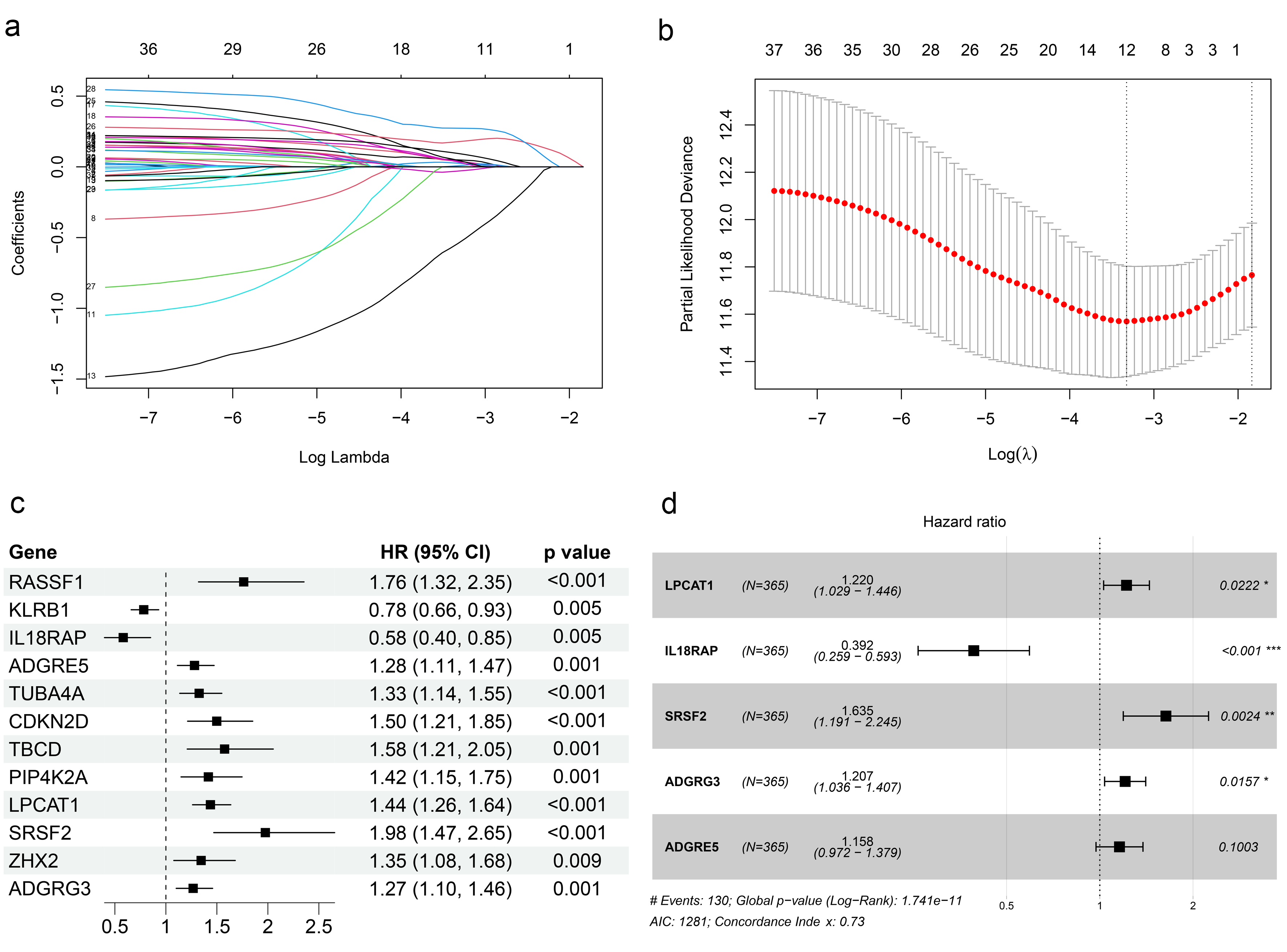

Supplement: Supplementary Figure 2 — Construction of NK cell marker genes-related prognostic signature in TCGA cohort. (A) LASSO coefficient profiles of the 37 prognostic genes. (B) In LASSO regression analysis, partial likelihood deviance plot showed cross-validation for tuning parameter screening. (C) Forest plots showing the results of the univariate Cox regression analysis between the 12 genes expression (identified by LASSO regression analysis) and OS in the TCGA cohort. (D) Step Multivariable Cox proportional hazards regression analysis of the 5 prognostic genes. [file Image_2.tif]

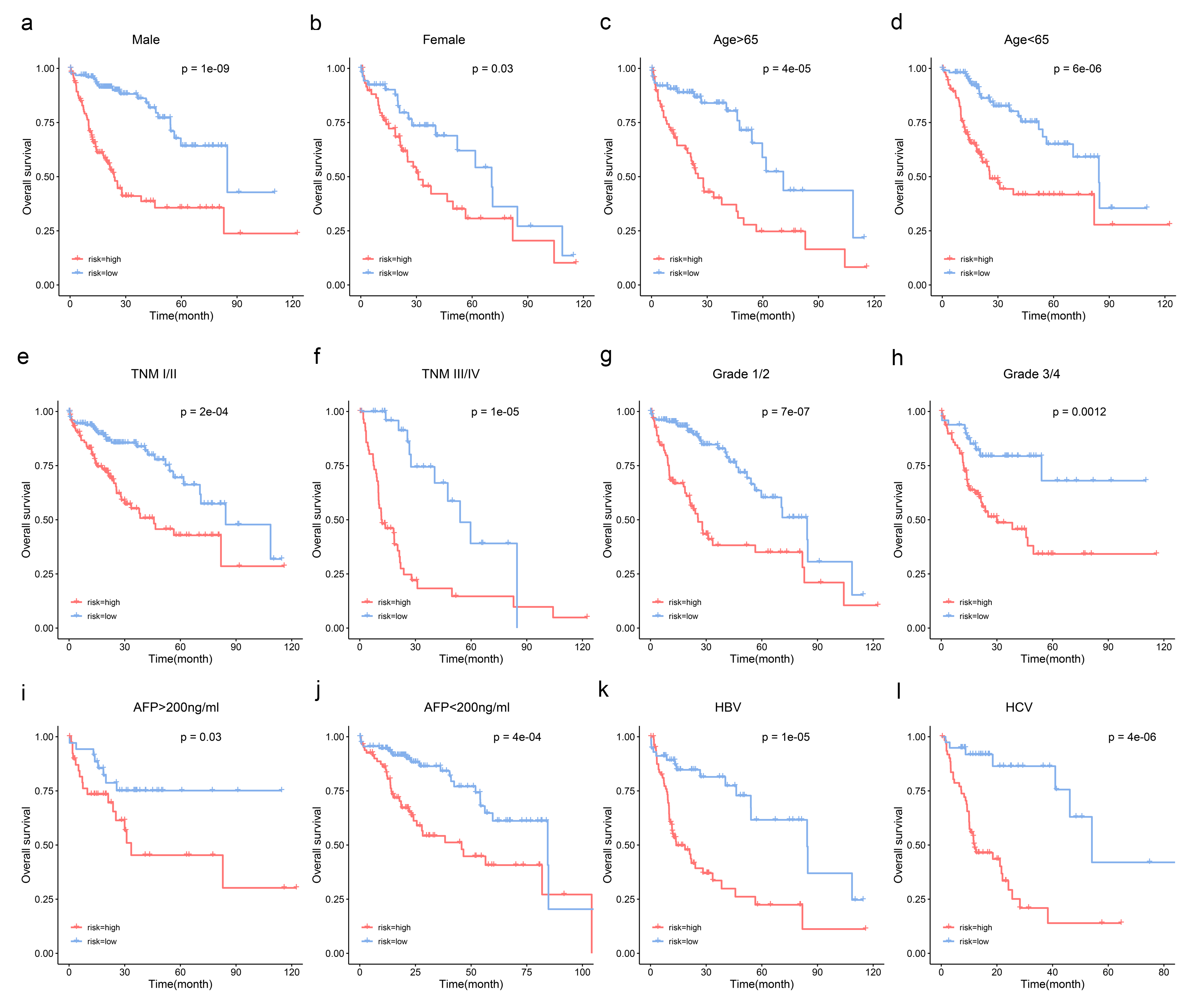

Supplement: Supplementary Figure 3 — Validation of the prognostic performance of NKPS in different clinical subgroups. Kaplan–Meier curves of overall survival in male (A), female (B), young (C), old (D), TNM I/II (E), TNM III/IV (F), early stage (G) and advanced stage (pathological grade 3/4) (H), AFP ≥200ng/ml (I), AFP<200 (J), HBV (K) and HCV (L) patients based on risk score in TCGA cohort. [file Image_3.tif]

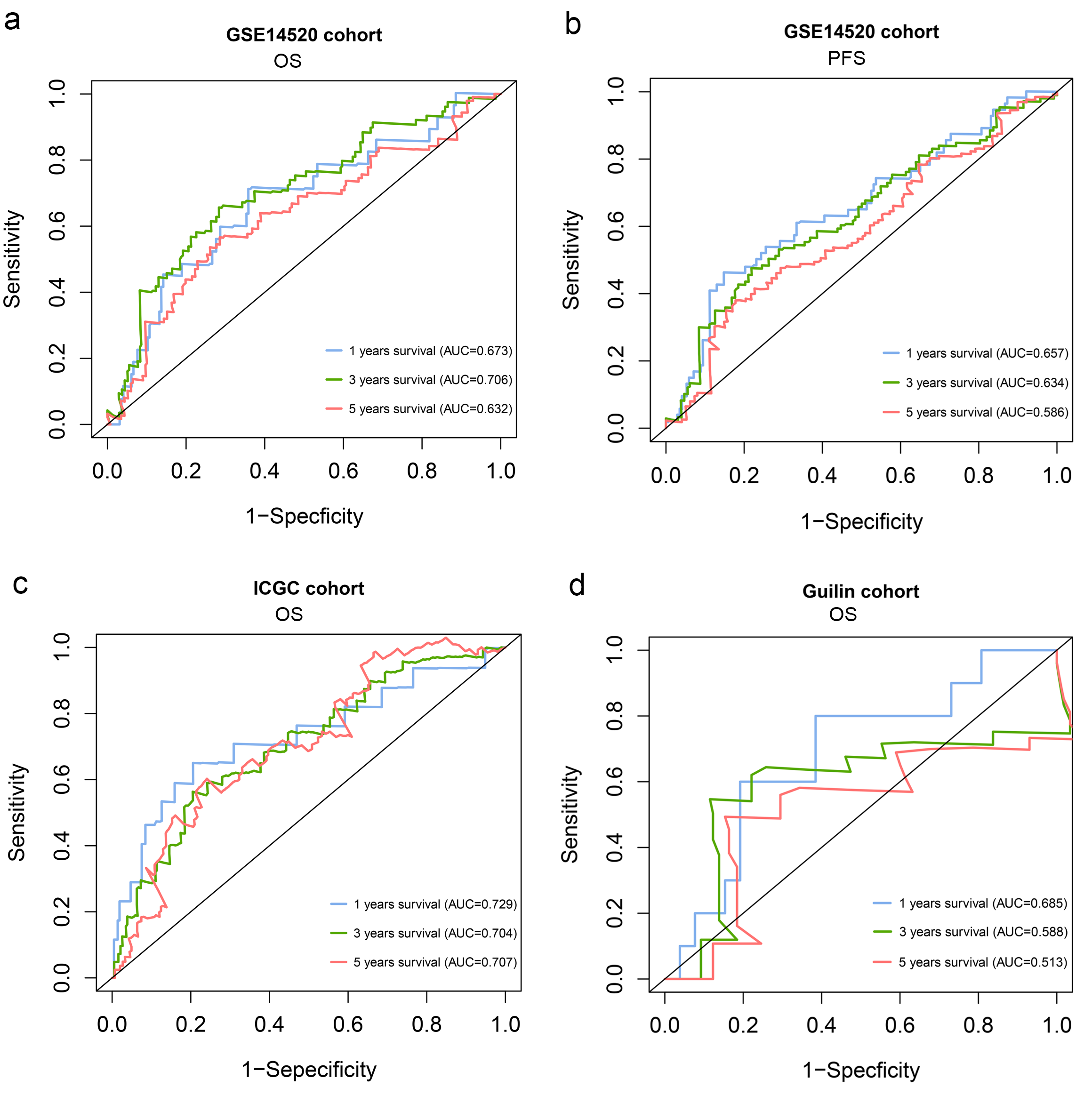

Supplement: Supplementary Figure 4 — Time-dependent ROC analysis of the NKPS for predicting the risk of death in different cohorts. (A) Time-dependent ROC analysis of the NKPS for predicting the risk of death at 1, 3, and 5 years in GSE14520 cohort. (B) Time-dependent ROC analysis of the NKPS for predicting the risk of disease progression at 1, 3, and 5 years in GSE14520 cohort. (C) Time-dependent ROC analysis of the NKPS for predicting the risk of death at 1, 3, and 5 years in ICGC cohort. (D) Time-dependent ROC analysis of the NKPS for predicting the risk of death at 1, 3, and 5 years in Guilin cohort. [file Image_4.tif]
