# Supplementary material for: Prognosis and therapeutic benefits prediction based on NK cell marker genes through single-cell RNA-seq with integrated bulk RNA-seq analysis for hepatocellular carcinoma
Source: Front Oncol. 2023 Jul 24;13:1208165. doi: 10.3389/fonc.2023.1208165 (PMC10406383; doi:10.3389/fonc.2023.1208165)
Supplement: Supplementary file 6 [file Table_1.docx]

Table S1. The sequences of primers used in Real‑time quantitative polymerase chain reaction

| Gene | Sequences of primers | |
| --- | --- | --- |
| LPCAT1 | Forward | ACATCCCGATCTGGGGAACT |
|  | Reverse | GGCCACTTTCCGTTGGACT |
| IL18RAP | Forward | TGCACAAAGTCCAGCGGTAA |
|  | Reverse | CAGCTCTGACTGTCCACGAA |
| SRSF2 | Forward | CCAAGTCCAAGTCCTCGTCG |
|  | Reverse | GCTTGCCGATACATCATTTTCT |
| ADGRG3 | Forward | CGAAGGGCCAAGAAACACCT |
|  | Reverse | CGTAGTTTAGCCAGTATCTCTGC |
| ADGRE5 | Forward | GGGACAAGAACGTCACTATGG |
|  | Reverse | GCCAGCAATGTCGTCATGT |
| GAPDH | Forward | CGGAGTCAACGGATTTGGTCGTAT |
|  | Reverse | AGCCTTCTCCATGGTGGTGAAGAC |

Table S2. correlation between NKPS and clinicopathological factors of HCC patients in the TCGA, ICGC, GSE14520 and Guilin cohorts.

|  | TCGA cohort | | | ICGC cohort | | | GSE14520 cohort | | | Guilin cohort | | |
| --- | --- | --- | --- | --- | --- | --- | --- | --- | --- | --- | --- | --- |
| Characteristics | **Low-risk** | **High-risk** | **P** | **Low-risk** | **High-risk** | **P** | **Low-risk** | **High-risk** | **P** | **Low-risk** | **High-risk** | **P** |
| Age  (Median, range) | 61  (16-85) | 61  (17-90) | 0.608 | 69  (31-86) | 69  (32-89) | 0.781 | 51  (25-74) | 50  (21-77) | 0.382 | 46  (32-73) | 45.5  (27-76) | 0.824 |
| Gender |  |  | 0.480 |  |  | 0.370 |  |  |  |  |  | 0.245 |
| Female | 56 | 63 |  | 34 | 27 |  | 17 | 13 | 0.574 | 6 | 2 |  |
| Male | 127 | 119 |  | 81 | 88 |  | 94 | 97 |  | 18 | 22 |  |
| TNM stage |  |  | <0.004 |  |  | 0.005 |  |  | 0.008 |  |  | 0.018 |
| I/II | 149 | 122 |  | 81 | 59 |  | 95 | 75 |  | 14 | 5 |  |
| III/IV | 32 | 59 |  | 34 | 56 |  | 15 | 34 |  | 10 | 19 |  |
| Unknown | 2 | 1 |  | 0 | 0 |  | 1 | 1 |  |  |  |  |
| Grade |  |  | 0.006 |  |  | 0.002 |  |  |  |  |  |  |
| 1/2 | 132 | 98 |  | 89 | 64 |  |  |  |  |  |  |  |
| 3/4 | 49 | 81 |  | 21 | 37 |  |  |  |  |  |  |  |
| Unknown | 2 | 3 |  | 5 | 14 |  |  |  |  |  |  |  |
| AFP |  |  | 0.141 |  |  |  |  |  | 0.001 |  |  |  |
| low | 115 | 85 |  |  |  |  | 73 | 45 |  |  |  |  |
| high | 35 | 40 |  |  |  |  | 37 | 63 |  |  |  |  |
| Unknown | 32 | 57 |  |  |  |  | 1 | 2 |  |  |  |  |
| BCLC stage |  |  |  |  |  |  |  |  | 0.002 |  |  |  |
| 0/A |  |  |  |  |  |  | 96 | 72 |  |  |  |  |
| B/C |  |  |  |  |  |  | 14 | 37 |  |  |  |  |
| unknown |  |  |  |  |  |  | 1 | 1 |  |  |  |  |

AFP: alpha-fetoprotein; TNM: tumor node metastasis; BCLC: Barcelona clinical liver cancer
